# Development of a triplex quantitative reverse transcription-polymerase chain reaction for the detection of porcine epidemic diarrhea virus, porcine transmissible gastroenteritis virus, and porcine rotavirus A

**DOI:** 10.3389/fmicb.2024.1390328

**Published:** 2024-05-10

**Authors:** Tingyu Luo, Kaili Li, Changwen Li, Changyou Xia, Caixia Gao

**Affiliations:** State Key Laboratory for Animal Disease Control and Prevention, Heilongjiang Provincial Key Laboratory of Laboratory Animal and Comparative Medicine, National Poultry Laboratory Animal Resource Center, Harbin Veterinary Research Institute, Chinese Academy of Agricultural Sciences, Harbin, China

**Keywords:** porcine epidemic diarrhea virus, porcine transmissible gastroenteritis virus, porcine rotavirus A, porcine enteric viruses, triplex real-time qRT-PCR

## Abstract

Porcine viral diarrhea is caused by many pathogens and can result in watery diarrhea, dehydration and death. Various detection methods, such as polymerase chain reaction (PCR) and real-time quantitative PCR (qPCR), have been widely used for molecular diagnosis. We developed a triplex real-time quantitative reverse transcription PCR (qRT-PCR) for the simultaneous detection of three RNA viruses potentially associated with porcine viral diarrhea: porcine epidemic diarrhea virus (PEDV), porcine transmissible gastroenteritis virus (TGEV), and porcine rotavirus A (PoRVA). The triplex qRT-PCR had *R*^2^ values of 0.999 for the standard curves of PEDV, TGEV and PoRVA. Importantly, the limits of detection for PEDV, TGEV and PoRVA were 10 copies/μL. The specificity test showed that the triplex qRT-PCR detected these three pathogens specifically, without cross-reaction with other pathogens. In addition, the approach had good repeatability and reproducibility, with intra-and inter-assay coefficients of variation <1%. Finally, this approach was evaluated for its practicality in the field using 256 anal swab samples. The positive rates of PEDV, TGEV and PoRVA were 2.73% (7/256), 3.91% (10/256) and 19.14% (49/256), respectively. The co-infection rate of two or more pathogens was 2.73% (7/256). The new triplex qRT-PCR was compared with the triplex RT-PCR recommended by the Chinese national standard (GB/T 36871-2018) and showed 100% agreement for PEDV and TGEV and 95.70% for PoRVA. Therefore, the triplex qRT-PCR provided an accurate and sensitive method for identifying three potential RNA viruses for porcine viral diarrhea that could be applied to diagnosis, surveillance and epidemiological investigation.

## 1 Introduction

The main pathogens causing diarrhea in piglets are porcine epidemic diarrhea virus (PEDV), porcine transmissible gastroenteritis virus (TGEV) and porcine rotavirus A (PoRVA) (Zhang et al., 2013; Monteagudo et al., 2022). Co-infection by these viruses is common in swine and poses a serious challenge for diarrhea control in swine farms (Zhang et al., 2019; Shi et al., 2021). PEDV is an enveloped, single-stranded, positive-sense RNA virus belonging to the genus *Coronavirus* in the family *Coronaviridae*. In 1978, researchers first isolated PEDV from the intestinal contents of pigs in the UK (Pensaert and de Bouck, 1978). PEDV has spread globally since then, causing watery diarrhea, vomiting, dehydration and death in pigs, resulting in severe economic losses for the swine industry (Wang et al., 2016). The PEDV genome is approximately 28 kb long and comprises seven open reading frames (ORFs). The *M* gene (ORF5) encodes the membrane protein M and has a relatively conserved sequence, which makes it a suitable molecular detection target for PEDV diagnosis (Kocherhans et al., 2001; Yang et al., 2014; Rasmussen et al., 2018). TGEV is also an RNA virus that belongs to the *Coronavirus* genus and *Coronaviridae* family. It was the first coronavirus identified in pigs and is responsible for porcine viral diarrhea. The genome of TGEV is approximately 28.6 kb in length and comprises nine major ORFs. The *N* gene (ORF6) encodes the capsid protein N and is relatively conserved in the TGEV genome (Eleouet et al., 1995; Yount et al., 2000). Another porcine enteric virus, PoRVA, is a non-enveloped double-stranded RNA virus belonging to the genus *Rotavirus* and family *Reoviridae*. It is one of the major pathogens responsible for severe diarrhea in piglets worldwide (Vlasova et al., 2017; Luo et al., 2023). The PoRVA genome is approximately 18.5 kb and has 11 double-stranded RNA segments. *NSP3* is a relatively conserved gene that plays a key role in viral replication and transcription, and is a common target gene for detecting PoRVA infection (Ghosh and Kobayashi, 2011).

Porcine viral diarrhea caused by these three enteric viruses poses serious health and economic threats to pig farming in China. It is also a challenge to the microbiological quality control of specific pathogen-free pigs for scientific research. To cope with the challenges of PEDV, TGEV and PoRVA, 34 standards have been publicly released in China so far, including 3 national standards, 6 agricultural industry and entry-exit inspection and quarantine industry standards, and 25 provincial local standards. These standards specify the detection methods for the three pathogens, such as reverse transcription polymerase chain reaction (RT-PCR), nested RT-PCR, single real-time quantitative RT-PCR (qRT-PCR) and duplex qRT-PCR. Among the 34 standards, a triplex RT-PCR technique was established only in the Chinese national standard (GB/T 36871-2018, 2018) for simultaneous detection and diagnosis of PEDV, TGEV and PoRVA. In addition, the duplex RT-PCR and duplex qRT-PCR techniques for differential diagnosis of dual infections by porcine viral diarrhea viruses were developed in some Chinese provincial local standards, e.g., Zhejiang provincial local standard (DB33/T 2254-2020, 2020) and Anhui provincial local standard (DB34/T 2795-2016, 2016). Porcine viral diarrhea is also severe worldwide, which has led to development of various pathogen detection techniques, such as triplex RT-PCR, nested RT-PCR and qRT-PCR (Huang et al., 2019; Chen et al., 2023). The accurate and rapid molecular diagnosis is essential for the prevention and control of the diseases caused by PEDV, TGEV and PoRVA. Therefore, it is necessary to establish a detection method with high specificity, sensitivity and efficiency.

Real-time qPCR is an accurate, sensitive, and rapid method for detecting and quantifying target genomes. Compared to conventional single qPCR, multiplex qPCR can simultaneously detect multiple target genes in a single reaction, showing many advantages such as high efficiency, throughput, and cost effective (Mackay, 2004; Yang et al., 2022). Advances in molecular biology techniques have led to widespread use of multiplex qPCR in clinical detection (Wang et al., 2020). In this study, we designed primers and probes based on the conserved fragments of PEDV *M* gene, TGEV *N* gene and PoRVA *NSP3* gene, and successfully developed a triplex qRT-PCR based on TaqMan probes. This method was highly sensitive and specific and did not cross-react with the genomes of other swine pathogens. It could be used for diagnosis, epidemiological investigation and microbiological quality control of specific pathogen-free pigs.

## 2 Materials and methods

### 2.1 Viral nucleic acids and clinical samples

The genomes (DNA or RNA) of PEDV, TGEV, PoRVA, pseudorabies virus (PRV), porcine circovirus type 2 (PCV2), porcine parvovirus (PPV), porcine deltacoronavirus (PDCoV), Seneca virus A (SVA), *Toxoplasma gondii*, *Leptospira interrogans*, *Mycoplasma hyopneumoniae*, *Mycoplasma hyorhinis*, *Haemophilus parasuis*, *Streptococcus suis*, *Pasteurella multocida* and *Actinobacillus pleuropneumoniae* were preserved by the State Key Laboratory for Animal Disease Control and Prevention of China or Heilongjiang Provincial Key Laboratory of Laboratory Animal and Comparative Medicine. In addition, 256 anal swab samples from pigs with or without clinical diarrhea were obtained from Animal Health Testing Center of Harbin Veterinary Research Institute, Chinese Academy of Agricultural Sciences. The Institutional Review Board of Harbin Veterinary Research Institute or the Regulations on the Administration of Laboratory Animals of China did not require the study to be reviewed or approved by an ethics committee because the samples were collected from animals that were already dead or euthanized for other purposes, and no additional harm or intervention was imposed on the animals.

### 2.2 Primers and TaqMan probes

**Figure 1:**
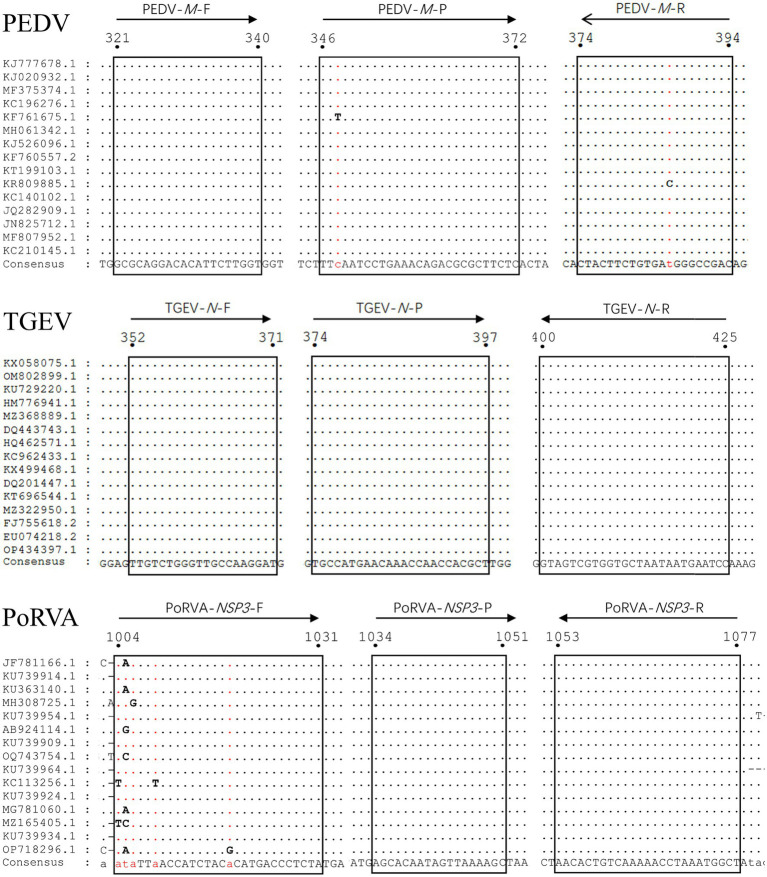
Alignment of sequences of reference viral strains collected from GenBank database. The locations of the primers/probes specific for PEDV *M* gene, TGEV *N* gene and PoRVA *NSP3* gene are shown. The positions of the partial nucleotide fragments are indicated by numbers.

To ensure the detection performance of the primers and probes used in the triplex qRT-PCR, *M* gene of PEDV genome, *N* gene of TGEV genome and *NSP3* gene of PoRVA genome were selected as the detection targets, based on their relative conservation. We obtained 86 PEDV *M* gene sequences, 46 TGEV *N* gene sequences and 20 PoRVA *NSP3* gene sequences from GenBank database. We used MegAlign to align them and determine the most conserved regions of each target gene. Using Primer Express 3.0.1, we designed primers and probes for the three viruses with the following conditions: primer length 18–30 bp, primer melting temperature (*T*_m_) 58–62°C, primer GC content 40–60%, product *T*_m_ 70–90°C and product size 70–150 bp. We ensured that the probe *T*_m_ value was higher than that of the primers. The specificity of the primers and probes was verified using the BLAST tool provided by the National Center for Biotechnology Information. For triplex detection, the probes for the three viral genes were labeled with different 5′-reporting dyes: Victoria Blue (VIC), Cyanine 5 (Cy5) and Fluorescein (FAM) and corresponding 3′-quenchers: Black Hole Quencher 1 (BHQ1), Black Hole Quencher 2 (BHQ2) and Minor Groove Binder (MGB). The triplex RT-PCR recommended by the Chinese national standard (GB/T 36871-2018, 2018) was used to verify the accuracy of the results for clinical samples. The details of the primers and probes are provided in Table 1. Figure 1 shows the locations of the triplex qRT-PCR primers and probes for PEDV, TGEV and PoRVA in different reference strains.

**Table 1:** Primers and probes used in this study.

Assays	Name	Sequence (5′–3′)	Product size (bp)	Reference
Triplex qRT-PCR	PEDV-*M*-F	GCGCAGGACACATTCTTGGT	74	This study
	PEDV-*M*-R	GTCGGCCCATCACAGAAGTAG		
	PEDV-*M*-P	VIC-TTCAATCCTGAAACAGACGCGCTTCTC-BHQ1		
	TGEV-*N*-F	TTGTCTGGGTTGCCAAGGAT	74	
	TGEV-*N*-R	GGATTCATTATTAGCACCACGACTAC		
	TGEV-*N*-P	Cy5-TGCCATGAACAAACCAACCACGCT-BHQ2		
	PoRVA-*NSP3-*F	AWATTAACCATCTACACATGACCCTCTA	74	
	PoRVA-*NSP3-*R	AGCCATTTAGGTTTTTGACAGTGTT		
	PoRVA-*NSP3-*P	FAM-AGCACAATAGTTAAAAGC-MGB		
Triplex RT-PCR	PEDV P1	TTCGGTTCTATTCCCGTTGATG	663	Chinese national standard (GB/T 36871-2018, 2018)
	PEDV P2	CCCATGAAGCACTTTCTCACTATC		
	TGEV P3	TTACAAACTCGCTATCGCATGG	528	
	TGEV P4	TCTTGTCACATCACCTTTACCTGC		
	PoRVA P5	CCCCGGTATTGAATATACCACAGT	333	
	PoRVA P6	TTTCTGTTGGCCACCCTTTAGT		

### 2.3 Preparation of standard plasmids

**Figure 2:**
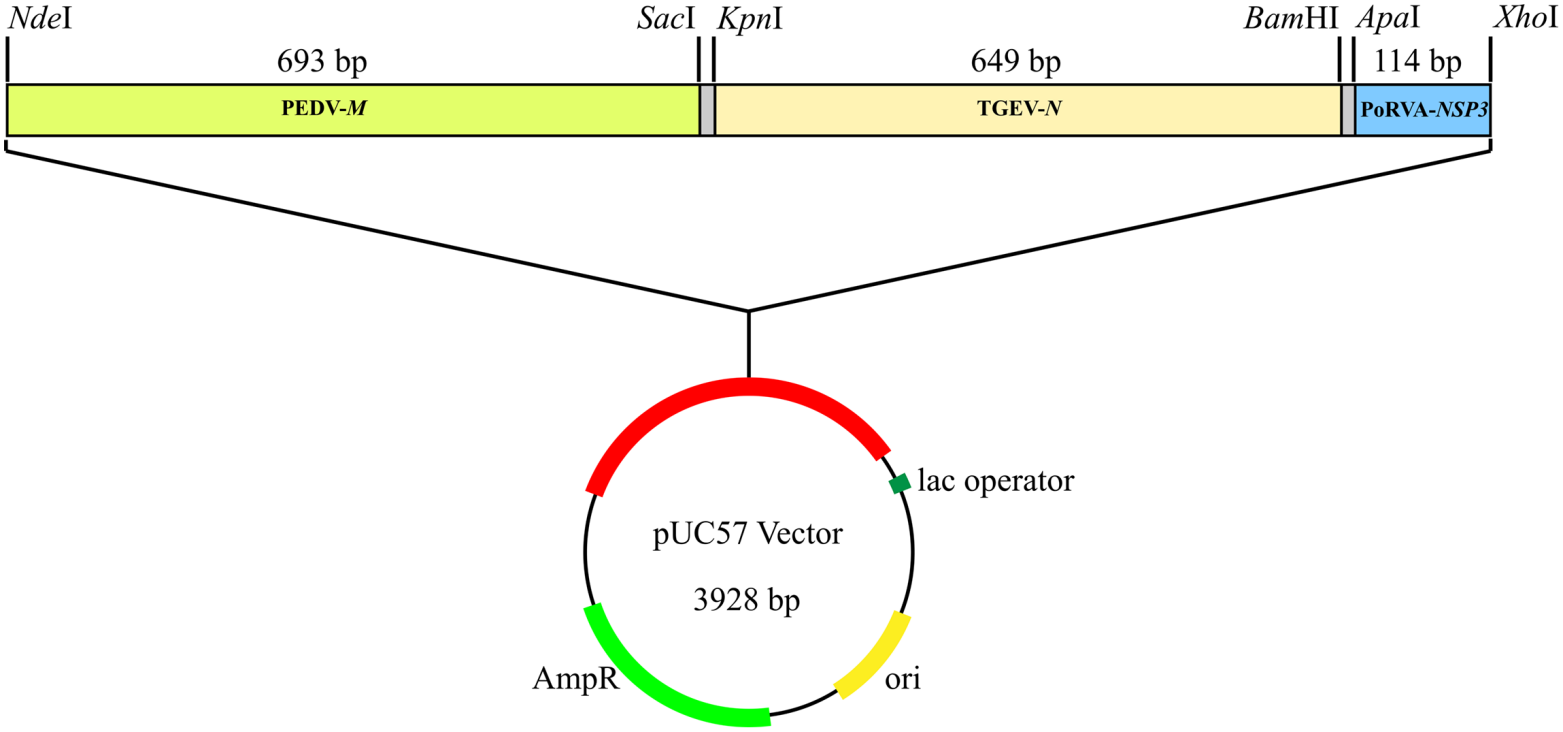
Standard plasmid containing three conserved gene fragments: PEDV *M* (693 bp), TGEV *N* (649 bp) and PoRVA *NSP3* (114 bp). Each fragment had specific restriction enzyme cutting sites at both ends: *Nde*I and *Sac*I for PEDV *M*; *Kpn*I and *Bam*HI for TGEV *N*; and *Apa*I and *Xho*I for PoRVA *NSP3*.

A synthetic gene fragment (PEDV-*M*-TGEV-*N*-PoRVA-*NSP3*) containing partial sequences of PEDV *M* gene, TGEV *N* gene and PoRVA *NSP3* gene was constructed in Sangon Biotech Co., Ltd. (Shanghai, China). This fragment was inserted into the pUC57 cloning vector, forming a standard plasmid (pUC57-PEDV *M* & TGEV *N* & PoRVA *NSP3*) for subsequent detection (Figure 2). The sequence of the synthetic gene fragment is shown in Supplementary Table S1. The plasmids were quantified by ultraviolet absorbance at 260 and 280 nm wavelengths using a NanoDrop spectrophotometer (Thermo Fisher Scientific, Waltham, MA, United States) and their copy number was calculated based on the size of the standard plasmid template using the following formula: copies/μL = (A260 (ng/μL) × 10^−9^ × 6.02 × 10^23^)/(DNA length × 650). The standard plasmids were serially diluted 10-fold to a concentration gradient of 10^8^–10^0^ copies/μL with EASY Dilution (TaKaRa, China, Dalian).

### 2.4 Optimization of the triplex qRT-PCR

The unoptimized triplex qRT-PCR consisted of 10 μL 2× One Step U^+^ Mix (Vazyme, China, Nanjing), 1 μL One Step U^+^ Enzyme Mix (Vazyme), 0.4 μL 50× ROX Reference Dye II, 0.4 μL each primer (final concentration of 200 nM), 0.2 μL each probe (final concentration of 100 nM), 4 μL template and 1.6 μL RNase-free water in a total volume of 20 μL. The amplification was performed on an ABI QuantStudio5 real-time PCR system (Thermo Fisher Scientific) with the following program: 55°C for 15 min, 95°C for 30 s; 40 cycles of 95°C for 10 s and 60°C for 30 s. The fluorescence signal was automatically collected at the end of each cycle. To optimize the reaction system, we explored different primer volumes (10 μM) and probe volumes (10 μM). A range of primer volumes (0.3–1.2 μL) was assessed to achieve final concentrations spanning 150–600 nM. Additionally, probe volumes were varied from 0.1 to 0.6 μL, covering a concentration range of 50–300 nM. Recombinant plasmids (10^7^ copies/μL) served as the detection template for optimization. Finally, the fluorescence intensity and cycle threshold (Ct) values of each primer and probe concentration were compared to determine the optimal volumes. The annealing temperature was also optimized by setting six gradients from 56 to 61°C and comparing the fluorescence intensity and Ct values of each gradient.

### 2.5 Establishment of standard curves for the triplex qRT-PCR

On the basis of the optimized reaction and protocol, three replicates of plasmid samples with serial dilutions from 10^8^ to 10 copies/μL were detected using the triplex qRT-PCR and subjected to linear regression between Ct values and the logarithm of plasmid copy numbers. Eight-point standard curves were established for PEDV, TGEV and PoRVA, including negative controls.

### 2.6 Specificity of the triplex qRT-PCR

To evaluate the specificity of the primer and probe sets, genomes (DNA or RNA) of PEDV, TGEV, PoRVA, PRV, PCV2, PPV, PDCoV, SVA, *T. gondii*, *L. interrogans*, *M. hyopneumoniae*, *M. hyorhinis*, *H. parasuis*, *S. suis*, *P. multocida* and *A. pleuropneumoniae* were tested using the triplex qRT-PCR. All nucleic acid samples were stored previously in our laboratory.

### 2.7 Sensitivity of the triplex qRT-PCR

For sensitivity assessment, standard plasmids were serially diluted 10-fold to final concentrations ranging from 10^8^ to 1 copies/μL. These dilutions were used as templates to determine the limit of detection (LoD) for triplex qRT-PCR and each reaction was repeated three times in a single test. We tested the standard plasmids at 100, 10 and 1 copies/μL 20 times to ensure the LoD accuracy. We set the LoD as the lowest concentration of standard plasmids that gave positive results in 85% of the replicates and marked it on the amplification curves. The threshold was set in the middle of the exponential amplification phase in the logarithmic view. A positive test result was defined as an exponential fluorescence curve that crossed the threshold within 35 cycles [(Ct) <35]. According to this definition, we calculated the positive rates at 100, 10 and 1 copies/μL of standard plasmids.

### 2.8 Repeatability and reproducibility of the triplex qRT-PCR

The repeatability (intra-assay precision) and reproducibility (inter-assay precision) of the developed triplex qRT-PCR were determined using standard plasmids at three different concentrations (10^6^, 10^4^ and 100 copies/μL). We analyzed each dilution in triplicate on the same day for intra-assay variability and in six independent experiments by two different operators on different days for inter-assay variability. The coefficient of variation (CV) of the Ct values of the samples at different concentrations was calculated in both intra-assay and inter-assay tests to estimate the repeatability and reproducibility.

### 2.9 Detection of clinical samples by the triplex qRT-PCR

Viral RNA was extracted from 256 anal swab samples using AxyPrep Body Fluid Viral DNA/RNA Miniprep Kit (Corning Life Sciences, China, Wujiang). The RNA samples were tested in triplicate by the optimized triplex qRT-PCR. Subsequently, the sample RNA was reverse transcribed into cDNA using PrimeScript^™^ RT Master Mix (Perfect Real Time) (TaKaRa) and detected by the triplex RT-PCR recommended by the Chinese national standard (GB/T 36871-2018, 2018), to validate the clinical performance of the developed triplex qRT-PCR assay. For the triplex RT-PCR, the reaction mixture (25 μL) contained 12.5 μL 2× Taq PCR Star Mix (Genstar, China, Beijing), 0.2 μL PEDV primers (final concentration of 80 nM), 0.4 μL TGEV primers (final concentration of 160 nM), 1 μL PoRVA primers (final concentration of 400 nM), 4 μL cDNA template and 5.3 μL RNase-free water. We performed the triplex RT-PCR with the following parameters: pre-denaturation at 94°C for 2 min, followed by 35 cycles of denaturation at 94°C for 30 s, annealing at 55°C for 30 s, extension at 72°C for 1 min, and final extension at 72°C for 10 min.

## 3 Results

### 3.1 Construction of the standard plasmid

A plasmid with three conserved gene fragments of PEDV *M* (693 bp), TGEV *N* (649 bp) and PoRVA *NSP3* (114 bp) was constructed, all containing their respective qRT-PCR amplicons. The plasmid was used as the standard for subsequent detection.

### 3.2 Optimization of the triplex qRT-PCR

**Figure 3:**
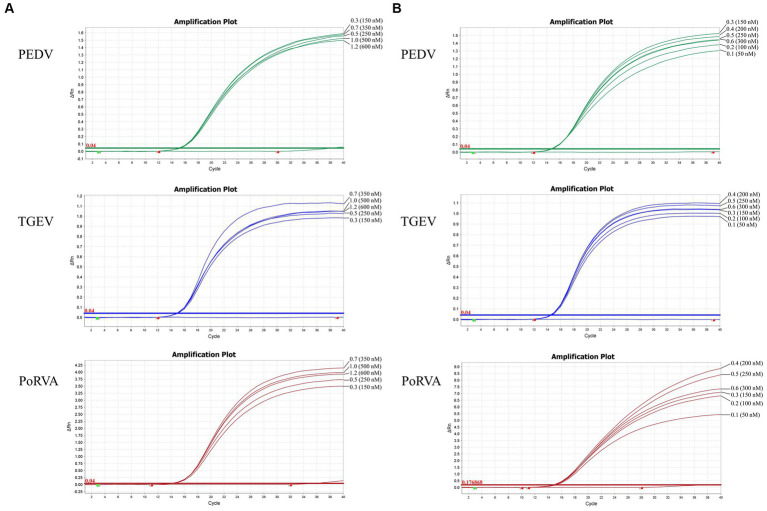
Optimization of the triplex qRT-PCR assay. **(A)** Optimization of primer volumes and the final concentrations in the reaction. The optimal volumes of forward and reverse primers were 0.7 μL for TGEV (final concentration: 350 nM) and PoRVA (final concentration: 350 nM), and 0.3 μL for PEDV (final concentration: 150 nM). **(B)** Optimization of probe volumes and the final concentrations in the reaction. The optimal volumes of probes were 0.4 μL for TGEV (final concentration: 200 nM) and PoRVA (final concentration: 200 nM), and 0.3 μL for PEDV (final concentration: 150 nM).

**Figure 4:**
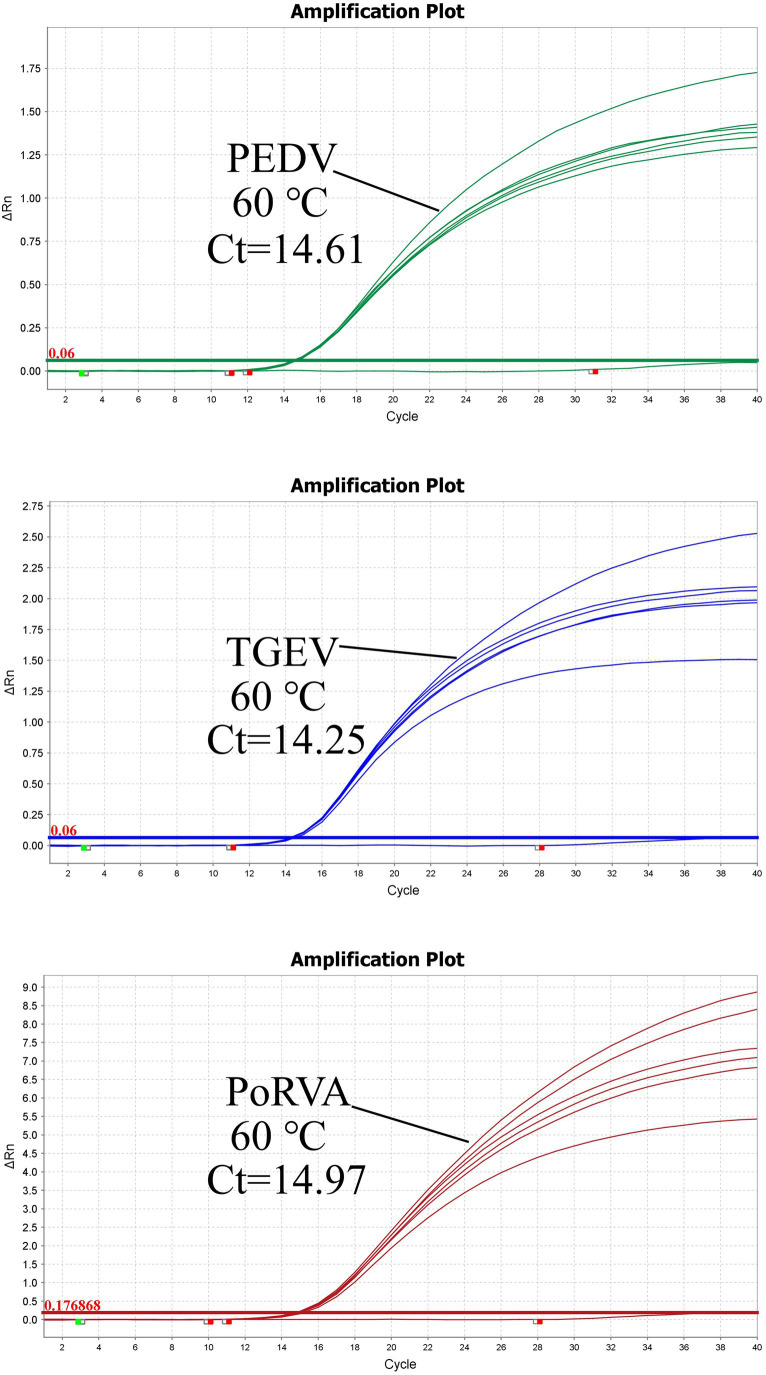
Triplex qRT-PCR amplification curves of different annealing temperatures. The optimal annealing temperature for triplex qRT-PCR was determined by measuring the amplification efficiency of the reaction at different *T*_m_ values. The highest amplification efficiency was achieved at 60°C.

Using the standard plasmid pUC57-PEDV *M* & TGEV *N* & PoRVA *NSP3* with the target fragments as the template, we optimized the reaction conditions of the triplex qRT-PCR. Orthogonal experiments determined the optimal annealing temperature and concentrations of primers and probes. For TGEV and PoRVA, the optimal volumes of probes and primers were 0.4 and 0.7 μL, respectively. For PEDV, both were 0.3 μL (Figure 3). The confirmed reaction system was listed in Table 2. The optimal annealing temperature was 60°C, which yielded the highest amplification efficiency (Figure 4).

**Table 2:** The reaction system of triplex qRT-PCR.

Reagent	Volume (μL) or concentration (nM)
2× One Step U^+^ Mix	10 μL
One Step U^+^ Enzyme Mix	1 μL
PEDV-*M*-F (10 μM)	0.3 μL (150 nM)
PEDV-*M*-R (10 μM)	0.3 μL (150 nM)
PEDV-*M*-P (10 μM)	0.3 μL (150 nM)
TGEV-*N*-F (10 μM)	0.7 μL (350 nM)
TGEV-*N*-R (10 μM)	0.7 μL (350 nM)
TGEV-*N*-P (10 μM)	0.4 μL (200 nM)
PoRVA-*NSP3-*F (10 μM)	0.7 μL (350 nM)
PoRVA-*NSP3-*R (10 μM)	0.7 μL (350 nM)
PoRVA-*NSP3-*P (10 μM)	0.4 μL (200 nM)
50× ROX Reference Dye II	0.4 μL
Template	4 μL
RNase-free water	0.1 μL
Total volume	20 μL

### 3.3 Establishment of the standard curve

**Figure 5:**
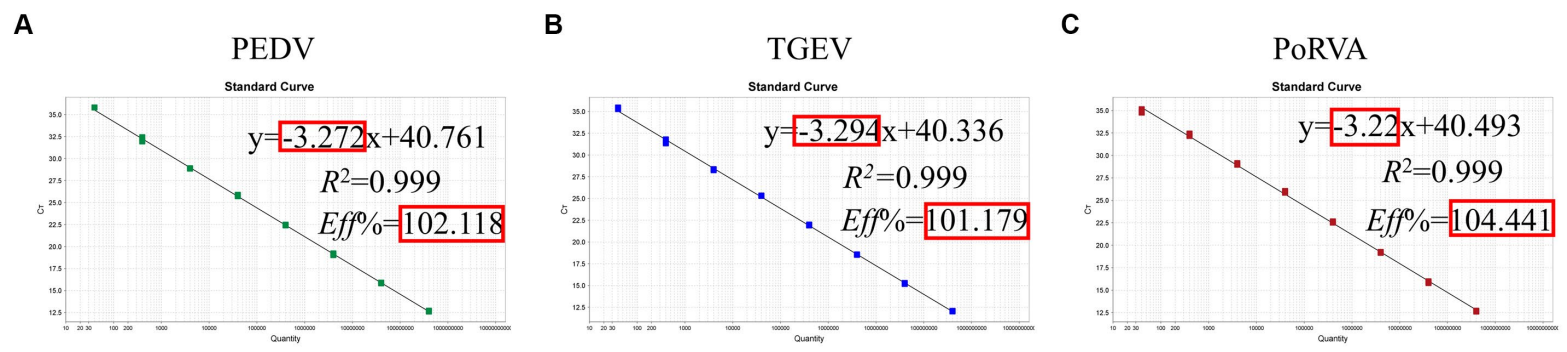
Standard curve for the triplex qRT-PCR assay. **(A)** Standard curve for PEDV *M* gene. **(B)** Standard curve for TGEV *N* gene. **(C)** Standard curve for PoRVA *NSP3* gene.

The standard plasmid was diluted in a 10-fold series and eight standard samples (10^8^–10 copies/μL) were selected as templates to establish the standard curve of the triplex qRT-PCR. Figure 5 shows the correlation coefficients (*R*^2^), equation slopes and amplification efficiencies (*E*) for each virus: PEDV, 0.999, −3.272 and 102.118%; TGEV, 0.999, −3.294 and 101.179%; and PoRVA, 0.999, −3.22 and 104.441%. The initial template and Ct value had a good linear relationship, as indicated by *R*^2^ and *E*.

### 3.4 Specificity of the triplex qRT-PCR

**Figure 6:**
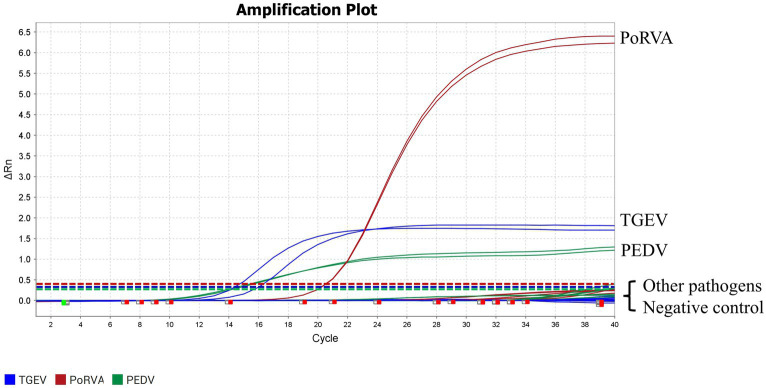
Specific amplification curves for the triplex qRT-PCR assay. Three fluorescent signals were monitored by triplex qRT-PCR. RNA of PEDV, TGEV and PoRVA was used as a positive control. No fluorescent signal was observed when genomes of other porcine pathogens were used as templates. The graph type was set in linear phase to simultaneously display the three different fluorescent signals (VIC, Cy5 and FAM) with distinct signal intensities. Other pathogens included PRV, PCV2, PPV, PDCoV, SVA, *T. gondii*, *L. interrogans*, *M. hyopneumoniae*, *M. hyorhinis*, *H. parasuis*, *S. suis*, *P. multocida* and *A. pleuropneumoniae.*

Genomic DNA or RNA of 16 porcine pathogens (PEDV, TGEV, PoRVA, PRV, PCV2, PPV, PDCoV, SVA, *T. gondii*, *L. interrogans*, *M. hyopneumoniae*, *M. hyorhinis*, *H. parasuis*, *S. suis*, *P. multocida* and *A. pleuropneumoniae*) was used as a template for the triplex qRT-PCR. Amplification curves were obtained for PEDV, TGEV and PoRVA but not for the other porcine pathogens (Figure 6). Therefore, the triplex qRT-PCR assay was specific for detection of PEDV, TGEV and PoRVA, and had no cross-reaction with other porcine pathogens.

### 3.5 Sensitivity of the triplex qRT-PCR

**Figure 7:**
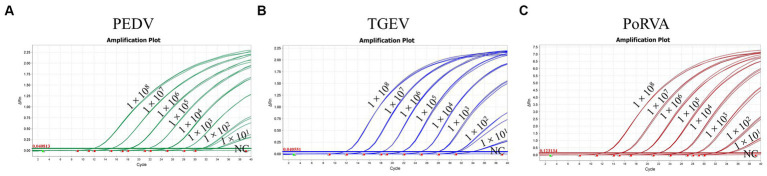
The sensitivity of the triplex qRT-PCR assay. **(A)** Sensitivity for PEDV *M* gene. **(B)** Sensitivity for TGEV *N* gene. **(C)** Sensitivity for PoRVA *NSP3* gene.

Different concentrations of standard plasmids were used as templates for the triplex qRT-PCR. Table 3 shows that a plasmid concentration of 100 copies/μL resulted in 100% positive detection rates for PEDV, TGEV and PoRVA. At 10 copies/μL, the positive detection rates were 100, 90 and 95% for PEDV, TGEV and PoRVA, respectively. At 1 copy/μL, the positive detection rates were 75, 5% and 0 for PEDV, TGEV and PoRVA, respectively. The LoD was defined as the lowest standard plasmid concentration with positive results in 85% of 20 replicates. Therefore, the triplex qRT-PCR showed high sensitivity, with a LoD of 10 copies/μL for PEDV, TGEV and PoRVA (Figure 7).

**Table 3:** Positive detection rate of 100 copies, 10 copies and 1 copy standard plasmids for 20 times.

Virus	Concentration	Positive number	Positive rate
PEDV	100 copies/μL	20	100%
	10 copies/μL	20	100%
	1 copy/μL	15	75%
TGEV	100 copies/μL	20	100%
	10 copies/μL	18	90%
	1 copy/μL	1	5%
PoRVA	100 copies/μL	20	100%
	10 copies/μL	19	95%
	1 copy/μL	0	0

### 3.6 Repeatability and reproducibility of the triplex qRT-PCR

Three concentrations of standard plasmids, 10^6^, 10^4^ and 100 copies/μL, were used to assess the repeatability and reproducibility of the triplex qRT-PCR. The intra-and inter-assay CVs were 0.08–0.79% and 0.37–0.83%, respectively (Table 4), which indicated good repeatability and reproducibility.

**Table 4:** Repeatability and reproducibility evaluation of the triplex qRT-PCR assay.

Virus	Concentration (copies/μL)	Intra-assay			Inter-assay		
		Mean Ct value	SD	CV (%)	Mean Ct value	SD	CV (%)
PEDV	10^6^	19.73	0.05	0.26%	19.63	0.16	0.83%
	10^4^	26.44	0.11	0.41%	26.34	0.19	0.73%
	10^2^	32.89	0.13	0.40%	32.62	0.27	0.82%
TGEV	10^6^	18.56	0.02	0.08%	18.51	0.08	0.42%
	10^4^	25.32	0.03	0.11%	25.24	0.10	0.40%
	10^2^	31.54	0.22	0.71%	31.23	0.26	0.83%
PoRVA	10^6^	19.12	0.10	0.51%	19.00	0.14	0.76%
	10^4^	25.82	0.08	0.31%	25.77	0.09	0.37%
	10^2^	32.14	0.26	0.79%	31.97	0.19	0.61%

### 3.7 Detection of clinical samples

A total of 256 porcine anal swab samples were tested using the triplex qRT-PCR. The positive rates for PEDV, TGEV and PoRVA were 2.73% (7/256), 3.91% (10/256) and 19.14% (49/256), respectively. To verify the accuracy of the method, the clinical samples were also tested by the triplex RT-PCR recommended by the Chinese national standard (GB/T 36871-2018, 2018). The triplex RT-PCR results showed that the positive rates for PEDV, TGEV and PoRVA were 2.73% (7/256), 3.91% (10/256) and 14.84% (38/256), respectively. Both methods detected seven samples co-infected with PEDV and PoRVA. The new triplex qRT-PCR had 100% (PEDV), 100% (TGEV) and 95.70% (PoRVA) agreement with the triplex RT-PCR, indicating that the new approach was accurate, reliable and more sensitive (Table 5).

**Table 5:** Detection of clinical samples by the triplex qRT-PCR and RT-PCR methods.

Virus	Triplex qRT-PCR (in this study)			Triplex RT-PCR Chinese national standard (GB/T 36871-2018, 2018)			Coincidence rate
	Sample number	Positive number	Positive rate	Sample number	Positive number	Positive rate	
PEDV	256	7	2.73%	256	7	2.73%	100%
TGEV	256	10	3.91%	256	10	3.91%	100%
PoRVA	256	49	19.14%	256	38	14.84%	95.70%
PEDV + TGEV	256	0	0	256	0	0	100%
PEDV + PoRVA	256	7	2.73%	256	7	2.73%	100%
TGEV + PoRVA	256	0	0	256	0	0	100%
PEDV + TGEV + PoRVA	256	0	0	256	0	0	100%

## 4 Discussion

PEDV, TGEV and PoRVA are porcine enteric RNA viruses that cause porcine viral diarrhea (Chen et al., 2010; Zhao et al., 2016; Zhang et al., 2017). Co-infections with various combinations and all three viruses are common in swine herds worldwide (Song et al., 2006; Liu et al., 2019; El-Tholoth et al., 2021). These co-infections severely compromise the herd immunity and result in an increased risk of secondary infections, higher piglet mortality and significant economic losses, and they are a major concern for the swine industry (Jung et al., 2008; Mesonero-Escuredo et al., 2018; Song et al., 2022).

Currently, the commonly used molecular detection techniques for PEDV, TGEV and PoRVA include RT-PCR, nested RT-PCR, qRT-PCR, reverse transcription loop-mediated isothermal amplification, reverse transcription recombinase-aided amplification, and CRISPR-Cas nucleic acid detection (Marthaler et al., 2014; Areekit et al., 2022; Wu et al., 2022; Lazov et al., 2023; Xia et al., 2024). RT-PCR and nested RT-PCR are not capable of quantitative analysis, and their operation is cumbersome, time-consuming and less sensitive. Isothermal amplification techniques, including reverse transcription loop-mediated isothermal amplification and reverse transcription recombinase-aided amplification, are prone to false-positive results. CRISPR-Cas nucleic acid detection is expensive and not suitable for large-scale detection, and its multiplex technology is not yet matured. qRT-PCR is a highly specific and sensitive method for quantifying trace amounts of RNA in samples and is the most practical technique for the detection of viral RNA. It displays the results as fluorescent signals, which are easy to interpret. In particular, the multiplex qRT-PCR technique can detect multiple target genes of various pathogens in a single-tube reaction, using specific primers and probes with different fluorescent labels. Researchers have established some multiplex qRT-PCR detection methods for pathogens related to porcine viral diarrhea (Han et al., 2019; Huang et al., 2019; Jia et al., 2019). The detection method using SYBR Green fluorescent dye requires validation of product specificity through melting curve analysis. However, in practical applications, issues such as false-positive signals, dye redistribution, and low sensitivity can affect the reliability of detection results. However, the one-step TaqMan probe-based multiplex qRT-PCR method allows simultaneous detection of various RNA viruses without prior reverse transcription. In practical applications, it is easier to operate and has strong practicality for daily monitoring of pig diseases.

In this study, we designed three pairs of virus-gene-specific primers and corresponding probes for one-step triplex qRT-PCR, which can simultaneously detect PEDV, TGEV and PoRVA in one tube. We inserted three gene fragments into the same vector to generate a standard plasmid containing three gene targets for the triplex qRT-PCR, rather than using a mixture of three standard plasmids. This approach reduced the preparation cost of standard plasmids and minimized the systematic errors from adding three different plasmids. The sensitivity test revealed a LoD of 10 copies/μL for each pathogen. A strong linear correlation between Ct values and standard copy numbers was demonstrated by the standard curve plots. The primer and probe sequences used in the detection method were highly specific, and the fluorescent dyes VIC, Cy5 and FAM did not interfere with each other. Thus, PEDV, TGEV and PoRVA were accurately detected without cross-reaction with other swine pathogens (PRV, PCV2, PPV, PDCoV, SVA, *T. gondii*, *L. interrogans*, *M. hyopneumoniae*, *M. hyorhinis*, *H. parasuis*, *S. suis*, *P. multocida* and *A. pleuropneumoniae*). Furthermore, we tested 256 porcine anal swab samples with the developed triplex qRT-PCR method to verify its practicality and usefulness in clinical samples. The results indicated that PEDV, TGEV and PoRVA were detected in 7 (2.73%), 10 (3.91%) and 49 (19.14%) samples, respectively. This suggested that PEDV, TGEV and PoRVA persisted in pig herds. Co-infection with two or more of PEDV, TGEV and PoRVA was also common, which can worsen immunosuppression and inflammation, increase the risk of secondary infection by other pathogens, and exacerbate these diseases. This was supported by the detection of seven samples that were co-infected with PEDV and PoRVA in 256 clinical samples. We also tested 256 samples with the triplex RT-PCR detection method recommended by the Chinese national standard (GB/T 36871-2018, 2018). The results showed that consistency rates of 100% (PEDV), 100% (TGEV) and 95.70% (PoRVA) between the two methods. The sensitivity of the triplex qRT-PCR was significantly higher than that of triplex RT-PCR.

## 5 Conclusion

We developed a triplex qRT-PCR for simultaneous and differential detection of PEDV, TGEV and PoRVA. This new method is cost-effective, efficient and user-friendly. It can obtain results within an hour regardless of the number of samples to be tested or diagnosed, whether for routine screening or temporary diagnosis of these three pathogens in pig herds. It provides a reliable detection technique for accurate diagnosis, epidemiological investigation and microbiological quality control of laboratory pigs.

## Data availability statement

The sequence presented in the study is showed in Supplementary Table S1, further inquiries can be directed to the corresponding authors.

## Ethics statement

The manuscript presents research on animals that do not require ethical approval for their study.

## Author contributions

TL: Investigation, Methodology, Writing – original draft. KL: Methodology, Validation, Writing – original draft. CL: Investigation, Project administration, Writing – original draft. CX: Project administration, Supervision, Writing – original draft. CG: Funding acquisition, Project administration, Supervision, Writing – review & editing.
