# Supplementary material for: Development of a triplex quantitative reverse transcription-polymerase chain reaction for the detection of porcine epidemic diarrhea virus, porcine transmissible gastroenteritis virus, and porcine rotavirus A
Source: Front Microbiol. 2024 May 10;15:1390328. doi: 10.3389/fmicb.2024.1390328 (PMC11117717; doi:10.3389/fmicb.2024.1390328)
Supplement: Supplementary file 1 [file Table_1.docx]

**SUPPLEMENTARY MATERIALS**

**Supplementary Table S1.** Synthetic gene fragment sequences used in this study

| Name | Sequence (5' - 3') | Size (bp) |
| --- | --- | --- |
| PEDV-*M*-TGEV-*N*-PoRVA-*NSP3* | catatgATGTCTAACGGTTCTATTCCCGTTGATGAGGTGATAACACCAATGATTCAACACCTTAGAAACTGGAATTTCACATGGAATATCATACTGACGATACTACTCGTAGTGCTTCAGTATGGCCATTACAAGTACTCTGCGTTCTTGTATGGTGTCAAGATGGCTATTCTATGGATACTTTGGCCTCTTGTGTTAGCACTGTCACTTTTTGACGCATGGGCTAGCTTTCAGGTCAATTGGGTCTTTTTTGCTTTCAGCATCCTTATGGCTTGCATCACTCTTATGCTGTGGATAATGTACTTTGTCAATAGCATTCGGTTGTGGCGCAGGACACATTCTTGGTGGTCTTTCAATCCTGAAACAGACGCGCTTCTCACTACTTCTGTGATGGGCCGACAGGTCTGCATTCCAGTGCTTGGAGCACCAACTGGTGTAACGCTAACACTCCTTAGTGGTACATTGCTTGTAGAGGGCTATAAGGTTGCTACTGGCGTACAGGTAAGTCAATTACCTAATTTCGTCACAGTCGCCAAGGCCACTACAACAATTGTCTACGGACGTGTTGGTCGTTCAGTCAATGCTTCATCTGGCACTGGTTGGGCTTTCTATGTCCGGTCCAAACACGGCGACTACTCAGCTGTGAGTAATCCGAGTTCGGTTCTCACAGATAGTGAGAAAGTGCTTCATTTAGTCTAAgagctcTCCAGGAACTggtaccGGAATAGACAAACTCGCTATCGCATGGTGAAGGGCCAACGTAAAGAGCTTCCTGAAAGGTGGTTCTTCTACTACTTAGGTACTGGACCTCATGCAGATGCCAAATTTAAAGATAAATTAGATGGAGTTGTCTGGGTTGCCAAGGATGGTGCCATGAACAAACCAACCACGCTTGGTAGTCGTGGTGCTAATAATGAATCCAAAGCTTTGAAATTCGATGGTAAAGTGCCAGGCGAATTTCAACTTGAAGTTAACCAGTCAAGGGACAATTCAAGGTCACGCTCTCAATCTAGATCTCGGTCTAGAAACAGATCTCAATCTAGAGGCAGGCAACAATCCAATAACAAGAAGGATGACAGTGTAGAACAAGCTGTTCTTGCCGCACTTAAAAAGTTAGGTGTTGACACAGAAAAACAACAGCAACGCTCTCGTTCTAAATCTAAAGAACGTAGTAACTCTAAAACAAGAGATACTACGCCTAAGAATGAAAACAAACACACCTGGAAGAGAACTGCAGGTAAAGGTGATGTGACAAGATTTTATGGAGCTAGAAGCAGTTCAGCCAATTTTGGTGACAGTGACCTCGTTGCCAATGGGAGCAGTGCCAAGCATTACCCACAATTGGCTGAATGTGTTCCATCTGTGTCTAGggatccCAACCACCGAgggcccGAATGTACCTACGAATAGTCACATAATATAACCAATATTAACCATCTACACATGACCCTCTATGAGCACAATAGTTAAAAGCTAACACTGTCAAAAACCTAAATGGCTATAGGGctcgag | 1512 |

Note: Lowercase letters represent the enzyme cutting sites at both ends of PEDV *M*, TGEV *N* and PoRVA *NSP3*.
